# Late presentation for HIV remains a major health issue in Spain: Results from a multicenter cohort study, 2004–2018

**DOI:** 10.1371/journal.pone.0249864

**Published:** 2021-04-21

**Authors:** Marta Rava, Lourdes Domínguez-Domínguez, Otilia Bisbal, Luis Fernando López-Cortés, Carmen Busca, Antonio Antela, Patricia González-Ruano, Cristina Hernández, Josè-Antonio Iribarren, Rafael Rubio, Santiago Moreno, Inmaculada Jarrín, Cohort of the Spanish HIV/AIDS Research Network (CoRIS)

**Affiliations:** 1 Unit AIDS Research Network Cohort (CoRIS), National Center of Epidemiology (CNE), Health Institute Carlos III (ISCIII), Madrid, Spain; 2 12 de Octubre University Hospital, Madrid, Spain; 3 Virgen del Rocío University Hospital, Sevilla, Spain; 4 La Paz University Hospital, Madrid, Spain; 5 University Clinical Hospital of Santiago de Compostela, Santiago de Compostela, Spain; 6 Infanta Sofía University Hospital, Madrid, Spain; 7 Príncipe de Asturias University Hospital, Alcalá de Henares, Madrid, Spain; 8 Department of Infectious Diseases, University Hospital, IIS Biodonostia, San Sebastián, Spain; 9 Ramón y Cajal University Hospital, Madrid, Spain; Katholieke Universiteit Leuven Rega Institute for Medical Research, BELGIUM

## Abstract

**Objectives:**

With the purpose of reducing the well-known negative impact of late presentation (LP) on people living with HIV (PLWH), guidelines on early HIV diagnosis were published in 2014 in Spain, but since then no data on LP prevalence have been published. To estimate prevalence and risk factors of LP and to evaluate their impact on the development of clinical outcomes in the Cohort of the Spanish HIV/AIDS Research Network (CoRIS) during 2004–2018.

**Methods:**

CoRIS is an open prospective multicenter cohort of PLWH, adults, naive to ART at entry. LP was defined as HIV diagnosis with CD4 count ≤350 cells/μL or an AIDS defining event (ADE). Multivariable Poisson regression models were used to estimate both prevalence ratios (PR) for the association of potential risk factors with LP and Incidence rate ratios (IRRs) for its impact on the development of the composite endpoint (first ADE, first serious non-AIDS event [SNAE] or overall mortality).

**Results:**

14,876 individuals were included. Overall, LP prevalence in 2004–2018 was 44.6%. Risk factors for LP included older age, having been infected through injection drug use or heterosexual intercourse, low educational level and originating from non-European countries. LP was associated with an increased risk of the composite endpoint (IRR: 1.34; 95%CI 1.20, 1.50), ADE (1.39; 1.18, 1.64), SNAE (1.22; 1.01, 1.47) and mortality (1.71; 1.41, 2.08).

**Conclusions:**

LP remains a health problem in Spain, mainly among certain populations, and is associated with greater morbidity and mortality. Public policies should be implemented to expand screening and early diagnosis of HIV infection, for a focus on those at greatest risk of LP.

## Introduction

Late presentation (LP) of HIV infection represents an important barrier in achieving the UNAIDS goals to end AIDS epidemic by 2030. Despite multiple efforts to establish strategies to improve the early diagnosis of HIV infection, the prevalence of LP remains high (between 40–60% in developed countries [1–3] and higher in developing countries [4]) and has not decreased during the last years. In Spain, results from the most recent study within the Cohort of the Spanish HIV/AIDS Research Network (CoRIS) showed an overall LP prevalence of 46.9% from 2004 to 2013 [5]. According to the last treatment cascade estimates [6], 13% of people living with HIV in Spain are unaware of it. Of those diagnosed, 97.3% were on ART, of whom 90.4% had an undetectable viral load. In 2014 the Spanish Ministry of Health published national guidelines for promoting early HIV diagnosis [7]. They recommended offering HIV testing when there is an in case of indication or clinical suspicion of HIV infection or AIDS but also recommended routine offer of HIV screening. So far, there is no information on whether the implementation of these guidelines have had an impact on LP prevalence.

The implications of LP are widespread: increased risk of morbidity and mortality [8, 9], suboptimal virologic and immunologic effectiveness of antiretroviral therapy [10], increased risk of transmission due to lack of awareness of HIV serostatus [11, 12] as well as higher negative impact on healthcare costs [13]. However, while it is clear that LP carries a risk for the development of AIDS-related diseases and death, [5] its relationship with the development of non-AIDS diseases is less clear, based only on studies that relate poor immunologic status to its development. To the best of our knowledge, there are very few studies that directly evaluate LP as a risk factor for the development of non-AIDS events.

Therefore, the aims of this study are (i) to estimate the prevalence and associated risk factors of LP and late presentation with advanced disease (LPAD) and their changes over time in the period 2004–2018, and (ii) to estimate the impact of LP and LPAD on the development of clinical outcomes including AIDS-defining events (ADE), serious non-AIDS events (SNAE) and mortality in participants from the CoRIS cohort.

## Materials and methods

### Study population

CoRIS is an open, prospective, multicenter cohort of subjects with confirmed HIV infection, naïve to ART at study entry. Participants are recruited in 46 centers from 13 of the 17 autonomous regions in Spain from 2004-onwards. Administrative censoring date for these analyses was 30 November 2018. A complete description of CoRIS has been published in 2007 [14]. The study was approved by the Research Ethic Committee of the Instituto de Salud Carlos III and was conducted in accordance with the Declaration of Helsinki. All patients gave their written informed consent.

Briefly, CoRIS collects a minimum dataset which includes baseline and follow-up socio-demographic, immunologic and clinical data, including ART medication. Data are highly standardized and are submitted for periodic quality control procedures. Patients are followed-up periodically in accordance with routine clinical practice. All patients undergo blood collection for immunologic analysis, including CD4+ and CD8+ T lymphocyte quantification. Furthermore, all centers are invited to provide data on incident ADE and non-AIDS events, including non-AIDS–defining malignancies and cardiovascular, renal, liver, psychiatric, bone, and metabolic events.

The study population includes all CoRIS participants, aged ≥ 18 years recruited from 1 January 2004 to 30 November 2018 who had available information on CD4 count or ADE between 4 weeks before and 24 weeks after enrolment. For analyses on association of LP and LPAD with first occurrence of clinical events, individuals followed-up for less than six months were excluded from the relevant analysis. Individuals monitored in centers not providing data on NAEs were excluded for the relevant analyses.

### Definitions of late presentation and late presentation with advanced disease

Using the consensus definition [15], LP was defined as an HIV-diagnosis at a CD4 count below 350 cells/μL between 4 weeks before and 24 weeks after enrolment or with an AIDS-defining event within the first 24 weeks after enrolment, both conditions met before ART initiation. Participants with LPAD were a subgroup of those with LP, with an HIV diagnosis at a CD4 count <200 cells/μL between 4 weeks before and 24 weeks after enrolment.

### Health outcomes

The primary composite endpoint was first ADE, first SNAE or death from any cause occurred from 6 months after enrolment.

SNAE consisted of the following conditions: cardiovascular disease (myocardial infarction, angina, heart disease, transient ischemic attack, reversible ischemic deficit, stroke and peripheral arteriopathy) or death from cardiovascular disease, renal disease (end-stage renal disease, initiation of dialysis or renal transplantation) or death from renal disease, liver disease (ascites, gastrointestinal hemorrhage due to esophageal varices, hepatic encephalopathy, liver transplantation) or death from liver disease, non-AIDS-defining cancer or death from non-AIDS-defining cancer, and infectious-related deaths.

### Statistical methods

Variables were summarized as medians and interquartile ranges (IQR) when continuous, and as percentages when categorical. Multivariable Poisson regression models with robust standard errors estimates [16] were used to estimate prevalence ratios (PRs) and 95% confidence intervals (CI) for the association of potential risk factors for LP and LPAD: in all these models we compared participants with LP vs those without LP (non-LP, CD4 count ≥350 cells/μL and no AIDS-defining event) and participants with LPAD with those without LPAD (non-LPAD, CD4 count ≥200 cells/μL and no AIDS-defining event). Variables included were: a combined variable of gender and HIV transmission category (men who have sex with men [MSM], injection drug users [IDU], heterosexual men, heterosexual women and other/unknown), age at enrolment (<30, 30–49, ≥50 years), educational level (none or primary education only, secondary education, university, other/unknown) and region of origin (Europe, Sub-Saharan Africa [SSA], Latin America [LA], other/unknown). To assess whether risk factors for LP and LPAD had changed over time, interaction terms between each risk factor and the time-period (2004–2008, 2009–2012 and 2013–2018) were included in the multivariable models. The same model was used to obtain prevalence of LP and LPAD adjusted for a combined variable of gender and HIV transmission category, age at enrolment, educational level and region of origin.

Incidence rates for the composite endpoint, the first ADE, the first SNAE and death from any cause were calculated as the number of new cases occurred after six months from enrolment divided by the total person-years at risk from six months after enrolment until the first event, last follow-up visit or death, whichever came first. Incidence rate ratios (IRRs) and 95%CI for the association between LP and LPAD and the development of the composite endpoint and its components were estimated with multivariable Poisson regression models with person-time at risk as the offset variable and robust standard error estimates accounting for clusters between centers. All models were adjusted for the combined variable of gender and HIV transmission category, educational level, region of origin and age, presence of hepatitis C virus (HCV) antibodies (no, yes or unknown), presence of hepatitis B virus surface antigen (HBsAg, no, yes or unknown) and viral load (<10,000, 10,000–100,000, ≥ 100,000 copies/mL or unknown) at enrolment.

All statistical analyses were performed using R version 4.0 [17].

### Sensitivity analyses

We performed sensitivity analyses using different definitions of LP and LPAD as HIV-diagnosis at a CD4 <350 cells/μL (for LP) or <200 cells/μL (for LPAD) or with an ADE in the first 4, 12 or 48 weeks after enrolment.

## Results

Of the 15,509 antiretroviral naïve individuals enrolled in CoRIS until the 30th November 2018, 14,876 individuals were included (S1 Fig) of whom 12,652 (85.0%) were men, 9,182 (61.7%) were MSM, 3,742 (25.2%) had a university degree and 10,800 (72.6%) were from Europe, mostly from Spain (8,725, 58.7%). At enrolment, median age was 35.2 years (1st-3rd quartile: 28.9, 42.9), median CD4 count was 397 cells/μL (215, 592), 1,961 (13.2%) individuals had an AIDS diagnosis and 5,102 (34.3%) had a viral load ≥100,000 copies/mL (Table 1).

**Table 1:** Sociodemographic and clinical characteristics of the participants included, and prevalence of late presentation and late presentation with advanced disease according to their characteristics at enrolment, Spain, 2004–2018 (n = 14,876).

	Overall,	Late presentation,	Late presentation with advanced disease,
	N = 14,876	N = 6,636 (44.6%)	N = 3,931 (26.4%)
	[N (%)]	[N (row %)]	[N (row %)]
Sex			
Females	2,224 (15.0%)	1,176 (52.9%)	761 (34.2%)
Males	12,652 (85.0%)	5,460 (43.2%)	3,170 (25.1%)
Age (year)			
Median [1^st^, 3^rd^ quartile]	35.2 (28.9, 42.9)		
<30	3,720 (25.0%)	1,199 (32.2%)	507 (13.6%)
30–49	9,326 (62.7%)	4,269 (45.8%)	2,573 (27.6%)
≥50	1,830 (12.3%)	1,168 (63.8%)	851 (46.5%)
Transmission group			
MSM	9,182 (61.7%)	3,344 (36.4%)	1,685 (18.4%)
IDU	1,090 (7.3%)	658 (60.4%)	449 (41.2%)
Heterosexual women	1,894 (12.7%)	999 (52.7%)	643 (33.9%)
Heterosexual men	2,177 (14.6%)	1,297 (59.6%)	906 (41.6%)
Other/unknown	533 (3.6%)	64 (60.4%)	248 (46.5%)
Educational level			
None or primary education only	1,864 (12.5%)	1,103 (59.2%)	730 (39.2%)
Secondary education	6,801 (45.7%)	2,923 (43.0%)	1,758 (25.8%)
University	3,742 (25.2%)	1,349 (36.1%)	650 (17.4%)
Other/unknown	2,469 (16.6%)	1,261 (51.1%)	793 (32.1%)
Region of origin			
Europe	10,800 (72.6%)	4,609 (42.7%)	2,749 (25.5%)
Sub-Saharan Africa	685 (4.6%)	407 (59.4%)	251 (36.6%)
Latin America	3,083 (20.7%)	1,465 (47.5%)	822 (26.7%)
Other/unknown	308 (2.1%)	155 (50.3%)	109 (35.4%)
CD4 count, cells/μL			
Median [1st; 3rd quartile]	397 [215, 592]		
AIDS-defining event			
No	12,915 (86.8%)	4,675 (36.2%)	1,970 (15.3%)
Yes	1,961 (13.2%)	1,961 (100.0%)	1,961 (100.0%)
Viral load, copies/mL			
<10,000	3,076 (20.7%)	668 (21.7%)	259 (8.4%)
10,000–100,000	6,238 (41.9%)	2,320 (37.2%)	1,031 (16.5%)
≥100,000	5,102 (34.3%)	3,295 (64.6%)	2,348 (46.0%)
Unknown	460 (3.1%)	353 (76.7%)	293 (63.7%)
HCV antibodies			
No	11,676 (78.5%)	5,013 (42.9%)	2,868 (24.6%)
Yes	1,468 (9.9%)	845 (57.6%)	564 (38.4%)
Unknown	1,732 (11.6%)	778 (44.9%)	499 (28.8%)
HBsAg			
No	9,169 (61.6%)	3,856 (42.1%)	2,189 (23.9%)
Yes	3,955 (26.6%)	1,997 (50.5%)	1,236 (31.3%)
Unknown	1,752 (11.8%)	783 (44.7%)	506 (28.9%)
Year			
2004–2008	4,434 (29.8%)	2,296 (51.8%)	1,502 (33.9%)
2009–2012	4,168 (28.0%)	1,705 (40.9%)	947 (22.7%)
2013–2018	6,274 (42.2%)	2,635 (42.0%)	1,482 (23.6%)

MSM: men who have sex with men, IDU: injection drug users, HCV: hepatitis C virus, HBsAg: hepatitis B surface antigen.

### Late presentation and late presentation with advanced disease: Magnitude, trends and risk factors

**Fig 1:**
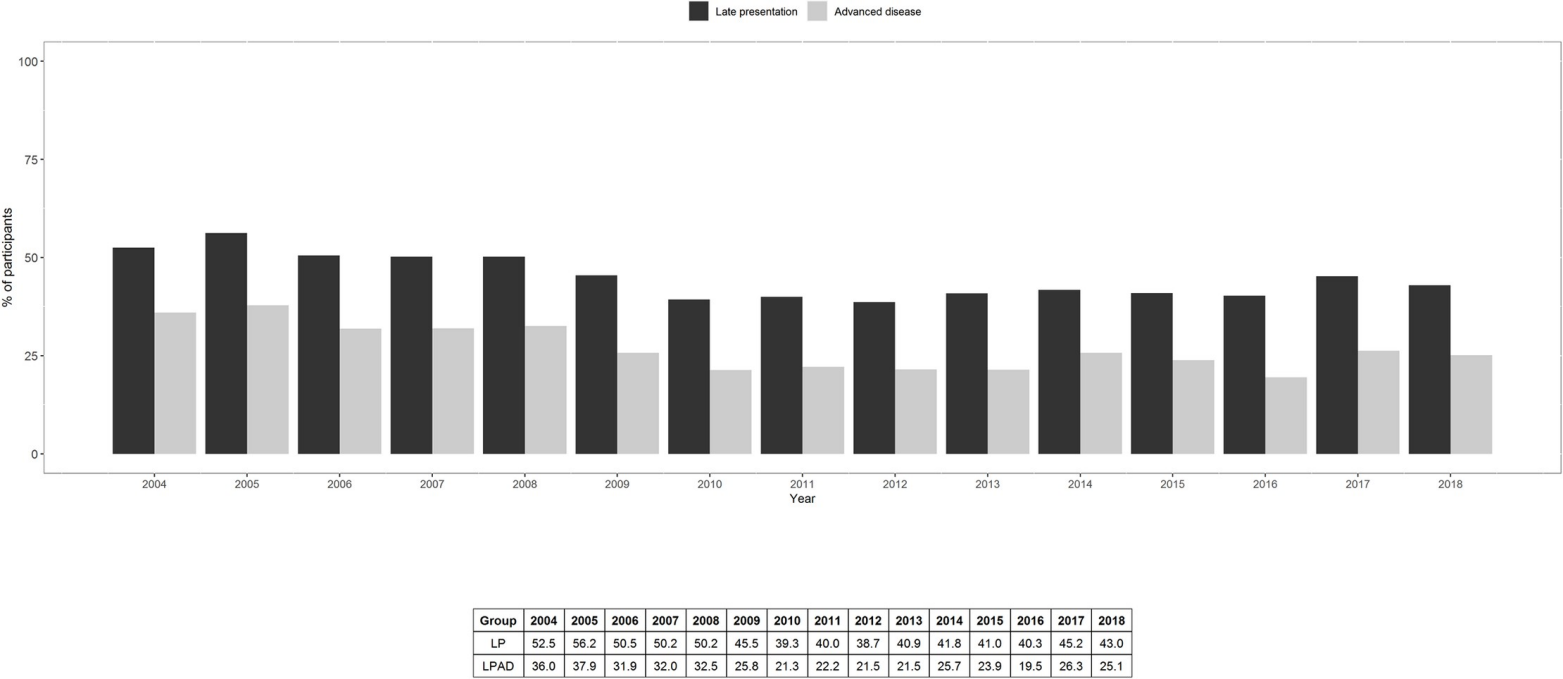
Annual prevalence of LP and LPAD, 2004–2018.

Total number of late presenters was 6,636 (prevalence 44.6%; 95%CI 43.8, 45.4) and of late presenters with advanced disease was 3,931 (26.4%; 95%CI 25.7, 27.1). Annual prevalence of LP and LPAD is shown in Fig 1. In 2004–2008 the prevalence of LP was 51.8% (95%CI 50.4, 53.3); it decreased to 40.9% (95%CI 39.4, 42.4) in 2009–2012 and remained stable at 42.0% (95%CI 40.8, 43.2) during 2013–2018 (p-value for trend <0.001). Likewise, the prevalence of LPAD decreased from 33.9% (95%CI 32.5, 35.3) in 2004–2008 to 22.7% (95%CI 21.5, 24.0) in 2009–2012 and remained stable with a modest increase up to 23.6% (95%CI 22.6, 24.7) during 2013–2018 (p-value for trend<0.001).

Among individuals older than 50 years, prevalence of LP was 63.8% and of LPAD was 46.5% (Table 1). Around 60% of IDU and of heterosexual men had LP and more than 40% had LPAD. Among persons originating from SSA, prevalence of LP was almost 60% and prevalence of LPAD was around 35%. Around 60% and of persons with primary or less educational level had LP and around 39% had LPAD.

Independent risk factors for LP compared to non-LP (Table 2) included older age (adjusted PR 1.36; 95%CI 1.30, 1.43 for 30–49 years and 1.77; 95%CI 1.65, 1.89 for ≥50 years vs <30 years) and transmission category, with IDU (aPR 1.49; 95%CI 1.33, 1.68), heterosexual men (aPR 1.41; 95%CI 1.27, 1.57) and heterosexual women (aPR 1.27; 95%CI 1.15, 1.41) having higher risks of LP compared to MSM. Other risk factors for LP were low educational level (aPR 1.31; 95%CI 1.20, 1.43 for primary education and 1.09; 95%CI 1.04, 1.14 for secondary education vs university) and region of origin with individuals originating from SSA (aPR 1.17; 95%CI 1.10, 1.25) and LA (aPR 1.23; 95%CI 1.16, 1.30) showing a higher risk of LP than Europeans. Similar risk factors were observed when we compared participants with LPAD with those without (Table 2).

**Table 2:** Independent risk factors associated with late presentation and late presentation with advanced disease, Spain, 2004–2018 (N = 14,876).

	Late presentation vs non-late presentation	Late presentation with advanced disease vs non-late presentation with advance disease
	Adjusted PR (95% CI) ^a^^,^ ^b^	Adjusted PR (95% CI) ^a^^,^ ^b^
Age (years):		
<30	1.00	1.00
30–49	1.36 (1.30, 1.43)	1.87 (1.71, 2.05)
≥50	1.77 (1.65, 1.89)	2.77 (2.52, 3.05)
Transmission category:		
MSM	1.00	1.00
IDU	1.49 (1.33, 1.68)	1.82 (1.53, 2.16)
Heterosexual women	1.27 (1.15, 1.41)	1.53 (1.31, 1.79)
Heterosexual men	1.41 (1.27, 1.57)	1.79 (1.53, 2.10)
Other/Unknown	1.50 (1.35, 1.67)	1.98 (1.68, 2.33)
Educational level:		
None or primary education only	1.31 (1.20, 1.43)	1.57 (1.39, 1.77)
Secondary education	1.09 (1.04, 1.14)	1.28 (1.18, 1.39)
Other/Unknown	1.21 (1.10, 1.33)	1.43 (1.24, 1.65)
University	1.00	1.00
Region of origin:		
Europe	1.00	1.00
Sub-Saharan Africa	1.17 (1.10, 1.25)	1.10 (0.97, 1.26)
Latin America	1.23 (1.16, 1.30)	1.23 (1.15, 1.32)
Other/Unknown	1.08 (0.97, 1.21)	1.21 (1.05, 1.40)

CI: confidence interval; MSM: men who have sex with men, IDU: injection drug users, PR: prevalence ratio.

^a^ Global p-values for each variable included in the model was <0.001.

^b^ Adjusted PR (95%CI): adjusted prevalence ratio and 95% CI obtained with multivariable Poisson regression models with robust standard error estimates adjusted for a combined variable of gender and HIV transmission category (MSM, IDU, heterosexual men, heterosexual women and other/unknown), age at enrolment (<30, 30–49, ≥50 years), educational level (None or primary education only, secondary education, university, other/unknown) and region of origin (Europe, Sub-Saharan Africa, Latin America, other/unknown).

**Fig 2:**
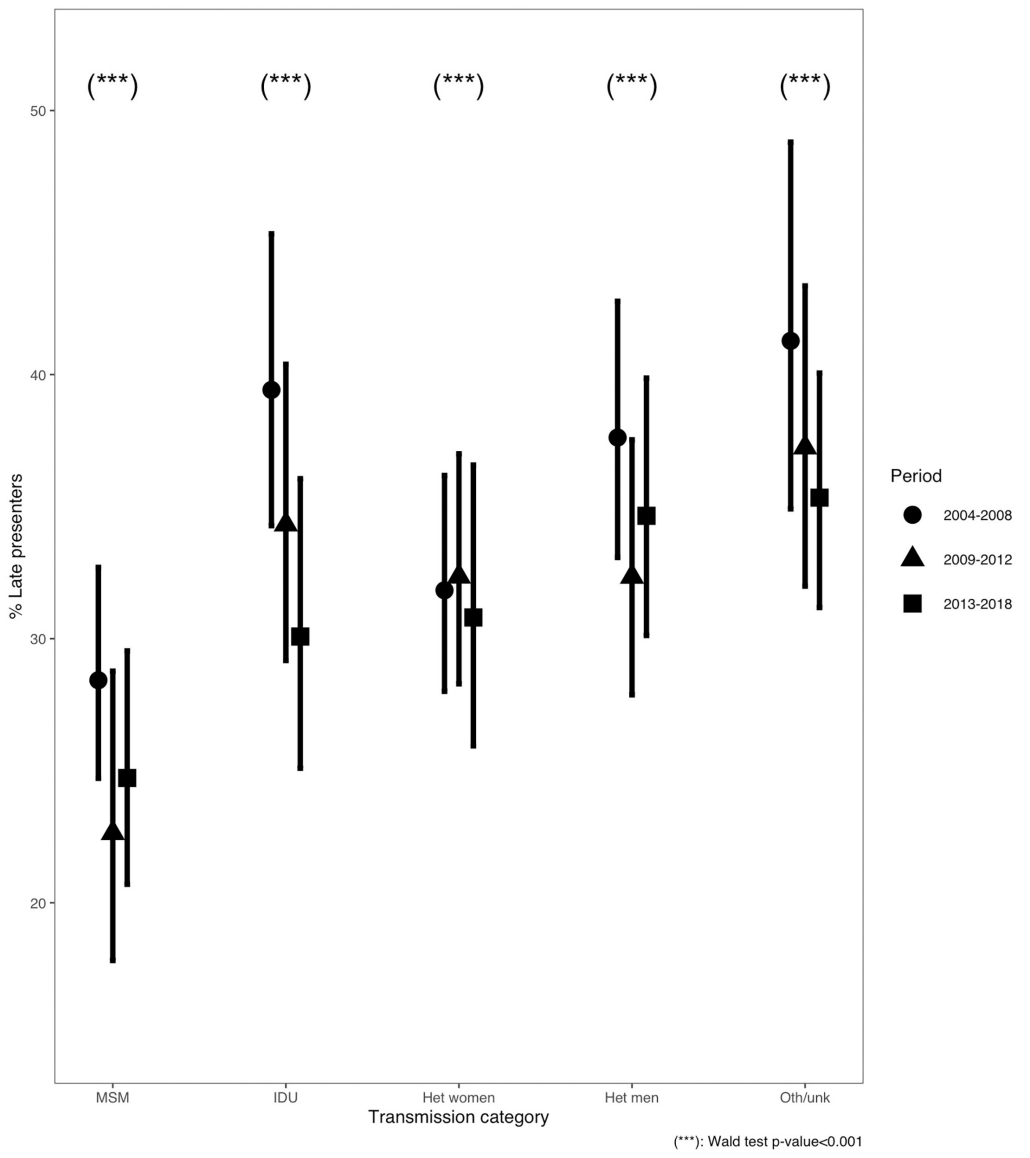
Adjusted prevalence of late presentation by transmission category and time-period.

Overall risk factors for LP and LPAD were confirmed during the three time periods considered (S1 Table). Nevertheless, adjusted LP prevalence were not homogeneous across transmission categories, as shown in Fig 2. Specifically, the adjusted prevalence of LP decreased from 28.4% in 2004–2008 to 22.6% in 2009–2012 in MSM and from 37.6% in 2004–2008 to 32.3% in 2009–2012 in heterosexual men, while adjusted LP prevalence in heterosexual women remained stable around 30%-33% during the whole 15-years period. Furthermore, while adjusted LP prevalence in MSM and heterosexual men, after an initial decrease from 2004–2008 to 2009–2012, remained stable during 2013–2018, adjusted LP prevalence in IDU continued to decrease from 39.4% in 2004–2008 to 34.3% in 2009–2012 to 30.1% in 2013–2018.

### Impact of late presentation and late presentation with advanced disease on morbidity and mortality

Of 14,876 participants, 13,031 (87.6%) had available follow-up data: 5,790 (87.3%) had LP and 3,412 (86.8%) had LPAD, with a total of 69,142 person-years (PY) of follow-up. Table 3 shows the number of participants experiencing each outcome, incidence rates (IR) per 100 PY of follow-up and incidence rate ratios for LP and LPAD.

**Table 3:** Impact of late presentation and late presentation with advanced disease on the composite endpoint, first ADE, first SNAE and overall mortality, Spain, 2004–2018, (N = 13,031 and 69,142 persons-year of follow-up).

	N of events	Follow-up (PYs)	Rate (95% CI) (per 100 PYs)	N of events	Follow-up (PYs)	Rate (95% CI) (per 100 PYs)	Unadjusted IRR (95% CI) ^a^^,^ ^b^	p-value	Adjusted IRR (95% CI) ^a^^,^ ^b^	p-value
	Non-late presenters			Late presenters			LP vs non-LP		LP vs non-LP	
Composite endpoint ^c^	435	36,046	1.21 (1.10, 1.33)	726	31,006	2.34 (2.17, 2.52)	1.95 (1.71, 2.22)	< 0.001	1.34 (1.20, 1.50)	< 0.001
ADE	177	34,840	0.51 (0.44, 0.59)	295	29,653	0.99 (0.88, 1.12)	1.95 (1.58, 2.42)	< 0.001	1.39 (1.18, 1.64)	< 0.001
SNAE ^c^^,^ ^d^	262	35,519	0.73 (0.65, 0.83)	406	30,769	1.32 (1.19, 1.45)	1.80 (1.53, 2.11)	< 0.001	1.22 (1.01, 1.47)	0.039
Cardiovascular event	85	35,752	0.24 (0.19, 0.29)	112	31,070	0.36 (0.30, 0.43)	1.52 (1.08, 2.13)	0.016	0.89 (0.63, 1.27)	0.534
Liver event	26	35,778	0.07 (0.05, 0.11)	58	31,178	0.19 (0.14, 0.24)	2.56 (1.73, 3.78)	< 0.001	1.74 (1.12, 2.71)	0.014
Kidney event	6	35,333	0.02 (0.01, 0.04)	9	30,561	0.03 (0.01, 0.06)	1.73 (0.55, 5.45)	0.346	0.84 (0.24, 2.92)	0.787
Neoplasm	150	34,367	0.44 (0.37, 0.51)	228	29,094	0.78 (0.69, 0.89)	1.80 (1.49, 2.17)	< 0.001	1.32 (1.08, 1.63)	0.007
Overall mortality	132	36,886	0.36 (0.30, 0.42)	321	32,253	1.00 (0.89, 1.11)	2.78 (2.27, 3.41)	< 0.001	1.71 (1.41, 2.08)	< 0.001
	Non-late presenters with advanced disease			Late presenters with advanced disease			LPAD vs non-LPAD		LPAD vs non-LPAD	
Composite endpoint ^c^	600	48,361	1.24 (1.14, 1.34)	561	18,691	3.00 (2.76, 3.26)	2.42 (2.08, 2.80)	< 0.001	1.66 (1.46, 1.89)	< 0.001
ADE	236	46,528	0.51 (0.44, 0.58)	236	17,966	1.31 (1.15, 1.49)	2.57 (2.06, 3.20)	< 0.001	1.89 (1.61, 2.23)	< 0.001
SNAE ^c^^,^ ^d^	360	47,473	0.76 (0.68, 0.84)	307	18,815	1.63 (1.45, 1.82)	2.15 (1.81, 2.56)	< 0.001	1.45 (1.18, 1.78)	< 0.001
Cardiovascular event	117	47,838	0.24 (0.20, 0.29)	80	18,983	0.42 (0.33, 0.52)	1.72 (1.29, 2.30)	< 0.001	0.93 (0.71, 1.21)	0.577
Liver event	38	47,873	0.08 (0.06, 0.11)	46	19,084	0.24 (0.18, 0.32)	3.04 (2.08, 4.43)	< 0.001	2.13 (1.30, 3.50)	0.003
Kidney event	8	47,271	0.02 (0.01, 0.03)	7	18,622	0.04 (0.02, 0.08)	2.22 (0.80, 6.16)	0.125	1.06 (0.32, 3.50)	0.918
Neoplasm	203	45,992	0.44 (0.38, 0.51)	175	17,469	1.00 (0.86, 1.16)	2.27 (1.87, 2.76)	< 0.001	1.70 (1.32, 2.19)	< 0.001
Overall mortality	197	49,456	0.40 (0.34, 0.46)	256	19,683	1.30 (1.15, 1.47)	3.27 (2.63, 4.06)	< 0.001	2.04 (1.64, 2.54)	< 0.001

ADE: AIDS defining event; CI: confidence interval; IRR: incidence rate ratio; LP: late presenters; LPAD: late presenters with advanced disease; PY: person-years; SNAE: serious non-AIDS event.

^a^ IRR (CI 95%) were estimated with Poisson regression models with person-years at risk as the offset variable and robust standard error estimates.

^**b**^ IRR estimates obtained after adjustment for a combined variable of gender and HIV transmission category (MSM, IDU, heterosexual men, heterosexual women and other/unknown), educational level (None or primary education only, secondary education, university, other/unknown), region of origin (Europe, Sub-Saharan Africa, Latin America, other/unknown), and age (<30, 30–49, ≥50 years), presence of HCV antibodies (no, yes or unknown), presence of HBsAg (no, yes or unknown) and viral load (<10,000, 10,000–100,000, ≥ 100,000 copies/mL or unknown) at enrolment.

^c^ Individuals who were monitored in centers not providing data on non-AIDS events were excluded.

^d^ The number of specific SNAE do not sum up because one individual can have multiple SNAE.

Overall, 435 non-late presenters and 726 late presenters experienced any of the events defined by the composite endpoint, with an IR of 1.21 (95%CI 1.10, 1.33) x100 PY in non-late presenters and of 2.34 (95%CI 2.17, 2.52) in late presenters, with an adjusted incidence rate ratio (aIRR) of 1.34 (95%CI 1.20, 1.50). IR for first ADE was almost double in participants with LP (IR: 0.99; 95%CI 0.88, 1.12) than in participants who were diagnosed early (IR: 0.51; 95%CI 0.44, 0.59), with an aIRR of 1.39 (95%CI 1.18, 1.64). 406 participants with LP experienced an SNAE with a rate of 1.32 (95%CI 1.19, 1.45) as did 262 participants non-late presenters with an IR of 0.73 (95%CI 0.65, 0.83), giving an aIRR of 1.22 (95%CI 1.01, 1.47). LP was associated with an increased risk of liver events (aIRR 1.74; 95%CI 1.12, 2.71), and neoplasms (aIRR 1.32; 95%CI 1.08, 1.63). Higher mortality rates were observed in late presenters (IR: 1.00; 95%CI 0.89, 1.11) than in non-late presenters (IR: 0.36; 95%CI 0.30, 0.42), with an aIRR of 1.71 (95%CI 1.41, 2.08).

561 participants with LPAD and 600 with non-LPAD experienced at least one event defined by the composite endpoint, with an IR of 3.00 (95%CI 2.76, 3.26) in participants with LPAD, an IR of 1.24 (95%CI 1.14, 1.34) in those with non-LPAD, and an aIRR of 1.66 (95%CI 1.46, 1.89). LPAD was associated with increased risk of developing an ADE (aIRR: 1.89; 95%CI 1.61, 2.23) and an SNAE (aIRR: 1.45; 95%CI 1.18, 1.78) and with increased risk of liver events (aIRR: 2.13; 95%CI 1.30, 3.50) and neoplasms (aIRR: 1.70; 95%CI 1.32, 2.19). Finally, participants with LPAD had higher mortality rates (IR: 1.30; 95%CI 1.15, 1.47) than non-late presenters with advanced disease (IR: 0.40; 95%CI 0.34, 0.46) with a two-fold increase in the risk of death (aIRR: 2.04; 95%CI 1.64, 2.54).

### Sensitivity analysis

When LP was defined as CD4 count <350 cells/μL or an AIDS diagnosis within 24 weeks of enrolment, the percentage of individuals who could not be classified as late presenters or non-late presenters was 6.1%, while it was around 4% when the window was modified to 4, 12 or 48 weeks (S2 Table). While considering different windows of time, similar prevalence was observed for LP (range: 44.6–45.0%) and for LPAD (range: 26.3–26.6%) (S2 Table). Highly consistent results were observed for the association between individual characteristics and LP and LPAD and on the risk of experiencing events of the composite endpoint and its components (S3 and S4 Tables).

## Discussion

This cohort study demonstrates that LP remains a major health issue in Spain. We observed a LP prevalence of 44.8% for the whole study period (2004–2018), and although a reduction was observed in 2009–2012, current data show a trend towards stabilization since then. We also observed a negative impact of both LP and LPAD on the occurrence of clinical outcomes, including ADE, SNAE and death.

We observed a similar LP prevalence in a previous CoRIS study [5], consistent with data from studies in the COHERE’s Europe region [18, 19] as well as in other European studies. (France 48% [1], Italy 54% [2], Belgium 44% [3], Germany 58.% [20], Switzerland 45% [21]). Similarly, the LPAD prevalence of 26% was close to the one observed in Belgium (24%, [3]), but slightly lower than the one described in the European region (33% [18, 19]) and in other nearby countries (France 29% [1], Italy 37% [2], Germany 36% [20]). Despite the decreasing trend from 2004–2008 to 2009–2012 and the publication in 2014 of the national guidelines on early HIV diagnosis [7], our estimates show a trend towards stabilization since then. The reasons why the prevalence of LP has stopped decreasing may be linked to the persistent low HIV testing frequency in Spain, which is around 20% [22]. This low rate may be due on one hand to the lack of awareness about HIV infection, the stigma as well as the lack of knowledge about health care services, and on the other hand to the lack of testing offer by the healthcare providers [22]. Promoting HIV testing should increase early HIV diagnosis in Spain.

The still high LP prevalence observed in our study may result from the influence of those factors classically associated with LP itself. As detected in other studies performed in geographically closely related regions [1, 2, 19, 20, 23, 24]. HIV acquisition mechanisms other than homosexual contact, as well as older age, low educational level and migrant status are consistently risk factors for LP and LPAD throughout the period. With respect to the HIV exposure group, a low self-perceived HIV-risk may be the reason why LP prevalence is higher among heterosexuals and those whose acquisition mechanism is unknown [25]. Furthermore, although IDU remains an important risk factor for LP, we observed that IDUs had the greatest decrease in LP prevalence, as observed in other studies [2, 5], which might be the consequence of an active engagement and linkage to care in centers designed for harm reduction programs in Spain. Consistent with data from CoRIS [5] and COHERE [26], we observed an increased risk of LP in participants with lower educational level. A higher educational level is associated with a more frequent access and use of health services; it may also be associated with increased health literacy and cognitive skills that improve health-related choices [26]. Furthermore, Fakoya I et al. [27] found that migrants had good access to primary care once they arrived in Europe and before their first positive HIV test, but language difficulties, lack of social support and cultural background (i.e.: the use of traditional medicine in the home country) were barriers to more frequent HIV testing and prevention after their arrival. [27, 28]. Díaz A et al. [29] demonstrated that risk of LP was stronger among migrants with low compared to high educational level in a Spanish sexually transmitted infections center.

Thus, we have found that the prevalence of late diagnosis remains high, especially in the most vulnerable group of patients, and that it has not decreased despite the publication of national guidelines on early HIV diagnosis [7]. This may be due in part to the difficulty of implementing recommendations as well as to the lack of resources [30]. For example, the quasi-experimental study ESTVIH [31] evaluated the feasibility of the application of different HIV diagnosis strategies in six Spanish primary care centers. Although healthcare workers expressed a high degree of interest in participating, only 26% of them recruited patients mainly because of the lack of time. Nevertheless, those trained to give pre and post-test counselling ordered the most tests [31]. A targeted strategy to identify persons with increased HIV risk has been proposed through the Drive 01 study [32]. The authors validated a structured questionnaire to assess HIV risk of exposure and indicator conditions specific to Spain, that predicted at 100% those individuals without infection and, if applied, it would make the test offer more efficient.

In terms of the clinical impact of LP, we found higher incidence rates for the composite endpoint and for ADE, SNAE and deaths both in patients presenting late compared to those with non-LP and in patients presenting with advanced disease, compared to those with non-LPAD. To the best of our knowledge, there is no other large cohort study which separately addresses ADE, SNAE and deaths.

Consistent with our results, researchers from the Dutch Athena cohort [33] and the MASTER cohort [34] observed an increased risk for the composite endpoint in participants with poor immune recovery.

As expected, the risk for ADE was higher in both the LP and the LPAD subgroups than in the non-LP and non-LPAD ones. Similarly, an Italian cohort [2] detected a reduction in LP mortality since the introduction of ART, which reflects the better management of frequent ADEs in these subgroups when the viral load is suppressed on ART.

Both LP and LPAD were associated with an increased risk for SNAE, compared with their counterparts. In the same cohort, Masía et al. [35] observed that the presence of ADE and the baseline CD4 count were risk factors for the development of SNAE and consequent overall mortality. Similarly, results from the D:A:D cohort showed that both last and nadir CD4 count were independent predictors of mortality due to ADE and SNAE [36]. Mocroft et al. [37] observed that a low CD4 count (<350 cells/μL) was associated with an increased risk of ADE or SNAE in patients from the EuroSIDA cohort. Possible mechanisms for the development of SNAE in LP [34] and LPAD [38] may be poor immune dysfunction, greater immune activation or persistent inflammation and oxidative stress. However, when interpreting these findings, it must be taken into account that there is great variability in SNAE definitions across studies, so results may not be directly comparable.

In terms of specific SNAE, consistent with our findings, Reekie et al. [39] found an increased incidence of some non-AIDS related cancers in patients with lower current CD4 count within the EuroSIDA Cohort. Impaired immunity could be certainly playing a role, as CD4 count depletion is associated with malignancy [40]. Moreover, CoRIS late presenter patients are older, and ageing-related immunosenescence is also linked with malignancies.

It may be argued that the association between LP and SNAE is confounded by age, which is a known risk factor for both conditions. For this reason, all our multivariable models are adjusted for age. Besides, Mocroft et al. [41] showed that the impact of high HIV progression risk (CD4 count ≤350 cells/μL, viral load ≥10,000 copies/mL) vs low (CD4 count ≥500 cells/μL, viral load <50 copies/mL) was greater in patients aged less than 30 years than in older patients. These findings highlight the important role of HIV infection in SNAE development even in groups with low risk of comorbidities development, such as young patients. Finally, we also observed an association between LP and LPAD and death from all causes. This finding is consistent with what was previously observed in the CoRIS cohort [5] especially for LPAD, with findings from Ingle et al [9].

The main strength of our study lies in being based on CoRIS, a large national cohort representative of the epidemiological situation of HIV-infected individuals in Spain. Furthermore, to the best of our knowledge, there is no other large cohort study which separately addresses ADE, SNAE and deaths, and the composite endpoint studied in relation to the LP and LPDA.

Our study has some limitations. First, we could not include all new HIV diagnoses in our analysis because 4% of cases lack information on CD4 count or ADE. Furthermore, LP prevalence may be overestimated because of a misclassification due to low CD4 count during acute HIV infection [42]. In addition, due to the low number of individuals experiencing some specific SNAE such as kidney events, these results should be interpreted with caution.

In conclusion, LP remains a major problem in Spain, with higher prevalence among certain populations, and is associated with greater morbidity and mortality. Public policies should be implemented to expand screening and early diagnosis of HIV infection for a focus on those at greatest risk of late presentation. As an example, targeted diagnostic programs could be run in emergency department, primary care and other medical centers; HIV testing could be considered as a priority in primary care protocols and information campaigns aimed at medical professionals could be carried out to publicize and improve the application of clinical guidelines.

## Supporting information

S1 Fig Flowchart of population selection. (DOCX)

S1 Table Independent risk factors for late presentation and late presentation with advanced disease by time-period. (DOCX)

S2 Table Prevalence of late presentation and late presentation with advanced disease when late presentation is defined as an HIV-diagnosis at a CD4 < 350/μL (or <200 /μL for advanced disease) or an AIDS-defining event within the 4, 12 or 48 weeks after enrolment by time-period (2004–2008, 2009–2012 and 2013–2018). (DOCX)

S3 Table Independent risk factors associated with late presentation and late presentation with advanced disease when late presentation is defined as an HIV-diagnosis at a CD4 <350 cells/μL (or <200 cells/μL for advanced disease) or an AIDS-defining event within the 4, 12 or 48 weeks after enrolment. (DOCX)

S4 Table Impact of late presentation and late presentation with advanced disease on the composite endpoint, first ADE, first SNAE and overall mortality when late presentation and late presentation with advanced disease are defined as participants with an HIV-diagnosis at a CD4 <350 cells/μL (or <200 cells/μL for advanced disease) or with an AIDS-defining event within the 4, 12 or 48 weeks after enrolment S4 Table. Impact of late presentation and late presentation with advanced disease on the composite endpoint, first AIDS event, first serious non-AIDS event and overall mortality when late presentation and late presentation with advanced disease are defined as participants with an HIV-diagnosis at a CD4 <350 cells/μL (or <200 cells/μL for advanced disease) or with an AIDS-defining event within the 4, 12 or 48 weeks after enrolment.
(DOCX)
