# Supplementary material for: Late presentation for HIV remains a major health issue in Spain: Results from a multicenter cohort study, 2004–2018
Source: PLoS One. 2021 Apr 21;16(4):e0249864. doi: 10.1371/journal.pone.0249864 (PMC8059864; doi:10.1371/journal.pone.0249864)
Supplement: S1 Fig — (DOCX) [file pone.0249864.s001.docx]

### S1 Fig. Flowchart of population selection
