# Supplementary material for: Late presentation for HIV remains a major health issue in Spain: Results from a multicenter cohort study, 2004–2018
Source: PLoS One. 2021 Apr 21;16(4):e0249864. doi: 10.1371/journal.pone.0249864 (PMC8059864; doi:10.1371/journal.pone.0249864)
Supplement: S1 Table — (DOCX) [file pone.0249864.s002.docx]

### S1 Table. Independent risk factors for late presentation and late presentation with advanced disease by time-period

|  | Late presenters | | | | Late presenters with advanced disease | | | |
| --- | --- | --- | --- | --- | --- | --- | --- | --- |
|  | 2004-2008 | 2009-2012 | 2013-2018 |  | 2004-2008 | 2009-2012 | 2013-2018 |  |
|  | Adjusted PR (95% CI) ^a^ | Adjusted PR (95% CI) ^a^ | Adjusted PR (95% CI) ^a^ | P-value for interaction ^b^ | Adjusted PR (95% CI) ^a^ | Adjusted PR (95% CI) ^a^ | Adjusted PR (95% CI) ^a^ | P-value for interaction ^b^ |
| Age (years): |  |  |  | 0.91 |  |  |  | 0.14 |
| <30 | 1.00 | 1.00 | 1.00 |  | 1.00 | 1.00 | 1.00 |  |
| 30-49 | 1.39 (1.29, 1.49) | 1.36 (1.26, 1.47) | 1.33 (1.23, 1.44) |  | 1.71 (1.53, 1.91) | 1.89 (1.47, 2.44) | 1.96 (1.71, 2.25) |  |
| ≥50 | 1.81 (1.67, 1.96) | 1.85 (1.63, 2.10) | 1.72 (1.53, 1.93) |  | 2.36 (2.03, 2.74) | 3.01 (2.29, 3.95) | 3.14 (2.68, 3.67) |  |
| Transmission category: |  |  |  | 0.00041 |  |  |  | 0.1 |
| MSM | 1.00 | 1.00 | 1.00 |  | 1.00 | 1.00 | 1.00 |  |
| IDU | 1.39 (1.27, 1.52) | 1.52 (1.22, 1.88) | 1.22 (1.01, 1.47) |  | 1.62 (1.39, 1.90) | 2.01 (1.49, 2.70) | 1.23 (0.89, 1.70) |  |
| Heterosexual women | 1.12 (1.02, 1.23) | 1.43 (1.20, 1.70) | 1.25 (1.11, 1.40) |  | 1.37 (1.21, 1.56) | 1.76 (1.34, 2.32) | 1.36 (1.14, 1.62) |  |
| Heterosexual men | 1.32 (1.21, 1.44) | 1.43 (1.19, 1.71) | 1.40 (1.25, 1.57) |  | 1.61 (1.38, 1.86) | 1.97 (1.57, 2.47) | 1.69 (1.43, 2.00) |  |
| Other/Unknown | 1.45 (1.28, 1.64) | 1.64 (1.36, 1.99) | 1.43 (1.27, 1.61) |  | 1.93 (1.57, 2.36) | 2.17 (1.66, 2.85) | 1.83 (1.51, 2.23) |  |
| Educational level: |  |  |  | 0.74 |  |  |  | 0.43 |
| None or primary education only | 1.28 (1.13, 1.44) | 1.34 (1.20, 1.50) | 1.31 (1.14, 1.50) |  | 1.49 (1.28, 1.74) | 1.81 (1.50, 2.19) | 1.46 (1.21, 1.76) |  |
| Secondary education | 1.09 (0.99, 1.21) | 1.08 (0.99, 1.19) | 1.04 (0.98, 1.11) |  | 1.25 (1.11, 1.42) | 1.35 (1.16, 1.56) | 1.18 (1.06, 1.31) |  |
| Other/Unknown | 1.19 (1.07, 1.32) | 1.24 (1.08, 1.43) | 1.21 (1.08, 1.35) |  | 1.32 (1.07, 1.64) | 1.47 (1.18, 1.84) | 1.46 (1.23, 1.72) |  |
| University | 1.00 | 1.00 | 1.00 |  | 1.00 | 1.00 | 1.00 |  |
| Region of origin: |  |  |  | 0.019 |  |  |  | 0.13 |
| Europe | 1.00 | 1.00 | 1.00 |  | 1.00 | 1.00 | 1.00 |  |
| Sub-Saharan Africa | 1.20 (1.11, 1.31) | 1.27 (1.12, 1.44) | 1.08 (0.94, 1.24) |  | 1.05 (0.89, 1.23) | 1.42 (1.17, 1.73) | 0.98 (0.77, 1.26) |  |
| Latin America | 1.16 (1.07, 1.26) | 1.24 (1.10, 1.39) | 1.28 (1.20, 1.37) |  | 1.20 (1.10, 1.30) | 1.33 (1.09, 1.63) | 1.25 (1.15, 1.37) |  |
| Other/Unknown | 1.02 (0.81, 1.27) | 1.27 (1.07, 1.52) | 1.09 (0.93, 1.28) |  | 0.95 (0.70, 1.29) | 1.59 (1.07, 2.37) | 1.31 (1.08, 1.59) |  |

CI: confidence interval; MSM: men who have sex with men, IDU: injection drug users, PR: prevalence ratio

^a^ Adjusted PR (95%CI): adjusted prevalence ratio and 95% CI obtained with multivariable Poisson regression models with robust standard error estimates adjusted for a combined variable of gender and HIV transmission category (MSM, IDU, heterosexual men, heterosexual women and other/unknown), age at enrolment (<30, 30-49, ≥50 years), educational level (None or primary education only, secondary education, university, other/unknown) and region of origin (Europe, Sub-Saharan Africa, Latin America, other/unknown).

^b^ P-value for interaction obtained by comparing the multivariate models with and without interaction term using a Wald test
