# Supplementary material for: Late presentation for HIV remains a major health issue in Spain: Results from a multicenter cohort study, 2004–2018
Source: PLoS One. 2021 Apr 21;16(4):e0249864. doi: 10.1371/journal.pone.0249864 (PMC8059864; doi:10.1371/journal.pone.0249864)
Supplement: S2 Table — (DOCX) [file pone.0249864.s003.docx]

**S2 Table. Prevalence of late presentation and late presentation with advanced disease when late presentation is defined as an HIV-diagnosis at a CD4 <350 cells/µL (or <200 cells/µL for advanced disease) or an AIDS-defining event within the 4, 12 or 48 weeks after enrolment by time-period (2004-2008, 2009-2012 and 2013-2018)**

| Time-window and time-period | Participants with data, N (%) | Prevalence of late presentation | Prevalence of late presentation with advance disease |
| --- | --- | --- | --- |
| 4 weeks | 14533 (93.9%) | 45.0 (44.2, 45.8) | 26.5 (25.8, 27.3) |
| 2004-2008 | 4278 | 52.5 (51.0; 54.1) | 34.3 (32.8; 35.7) |
| 2009-2012 | 4058 | 41.5 (40.0; 43.0) | 22.9 (21.6; 24.2) |
| 2013-2018 | 6197 | 42.2 (40.9; 43.4) | 23.6 (22.5; 24.7) |
| 12 weeks | 14794 (95.6%) | 44.6 (43.8, 45.5) | 26.3 (25.6, 27.1) |
| 2004-2008 | 4392 | 51.9 (50.4; 53.4) | 33.9 (32.5; 35.3) |
| 2009-2012 | 4144 | 41.0 (39.5; 42.5) | 22.6 (21.4; 23.9) |
| 2013-2018 | 6258 | 42.0 (40.8; 43.2) | 23.6 (22.5; 24.6) |
| 48 weeks | 14925 (96.4%) | 44.7 (43.9, 45.5) | 26.6 (25.9, 27.3) |
| 2004-2008 | 4459 | 51.8 (50.3; 53.3) | 34.0 (32.6; 35.4) |
| 2009-2012 | 4178 | 41.0 (39.5; 42.5) | 23.0 (21.7; 24.3) |
| 2013-2018 | 6288 | 42.0 (40.8; 43.3) | 23.7 (22.7; 24.8) |
