# Supplementary material for: Late presentation for HIV remains a major health issue in Spain: Results from a multicenter cohort study, 2004–2018
Source: PLoS One. 2021 Apr 21;16(4):e0249864. doi: 10.1371/journal.pone.0249864 (PMC8059864; doi:10.1371/journal.pone.0249864)
Supplement: S3 Table — (DOCX) [file pone.0249864.s004.docx]

##### **S3 Table. Independent risk factors associated with late presentation and late presentation with advanced disease when late presentation is defined as an HIV-diagnosis at a CD4 <350 cells/µL (or <200 cells/µL for advanced disease) or an AIDS-defining event within the 4, 12 or 48 weeks after enrolment.**

|  | 4 weeks |  | 12 weeks |  | 48 weeks |  |
| --- | --- | --- | --- | --- | --- | --- |
| Time-window | LP vs non-LP | LPAD vs non-LPAD | LP vs non-LP | LPAD vs non-LPAD | LP vs non-LP | LPAD vs non-LPAD |
|  | Adjusted PR (95% CI) ^a^ | Adjusted PR (95% CI) ^a^ | Adjusted PR (95% CI) ^a^ | Adjusted PR (95% CI) ^a^ | Adjusted PR (95% CI) ^a^ | Adjusted PR (95% CI) ^a^ |
| Age (years): |  |  |  |  |  |  |
| <30 | 1.00 | 1.00 | 1.00 | 1.00 | 1.00 | 1.00 |
| 30-49 | 1.36 (1.30, 1.42) | 1.86 (1.70, 2.03) | 1.36 (1.30, 1.42) | 1.86 (1.70, 2.04) | 1.36 (1.30, 1.42) | 1.87 (1.71, 2.05) |
| ≥50 | 1.76 (1.65, 1.88) | 2.74 (2.49, 3.01) | 1.76 (1.65, 1.88) | 2.76 (2.50, 3.03) | 1.76 (1.65, 1.88) | 2.77 (2.52, 3.05) |
| Transmission category: |  |  |  |  |  |  |
| MSM | 1.00 | 1.00 | 1.00 | 1.00 | 1.00 | 1.00 |
| IDU | 1.50 (1.33, 1.69) | 1.85 (1.56, 2.20) | 1.49 (1.33, 1.68) | 1.82 (1.54, 2.16) | 1.49 (1.33, 1.67) | 1.83 (1.54, 2.16) |
| Heterosexual women | 1.28 (1.15, 1.42) | 1.56 (1.33, 1.82) | 1.28 (1.15, 1.42) | 1.54 (1.32, 1.80) | 1.27 (1.15, 1.41) | 1.53 (1.31, 1.78) |
| Heterosexual men | 1.42 (1.28, 1.58) | 1.82 (1.56, 2.14) | 1.42 (1.28, 1.58) | 1.80 (1.54, 2.11) | 1.41 (1.27, 1.57) | 1.79 (1.53, 2.09) |
| Other/Unknown | 1.49 (1.34, 1.67) | 2.01 (1.70, 2.38) | 1.50 (1.35, 1.67) | 2.00 (1.69, 2.36) | 1.50 (1.34, 1.67) | 1.98 (1.67, 2.34) |
| Educational level: |  |  |  |  |  |  |
| None or primary education only | 1.30 (1.19, 1.42) | 1.54 (1.37, 1.74) | 1.31 (1.20, 1.43) | 1.55 (1.38, 1.75) | 1.30 (1.19, 1.42) | 1.54 (1.36, 1.74) |
| Secondary education | 1.09 (1.04, 1.14) | 1.27 (1.18, 1.37) | 1.08 (1.04, 1.14) | 1.27 (1.17, 1.38) | 1.08 (1.04, 1.13) | 1.27 (1.17, 1.38) |
| Other/Unknown | 1.22 (1.11, 1.34) | 1.44 (1.25, 1.66) | 1.21 (1.10, 1.33) | 1.43 (1.24, 1.64) | 1.21 (1.10, 1.32) | 1.41 (1.23, 1.63) |
| University | 1.00 | 1.00 | 1.00 | 1.00 | 1.00 | 1.00 |
| Region of origin: |  |  |  |  |  |  |
| Europe | 1.00 | 1.00 | 1.00 | 1.00 | 1.00 | 1.00 |
| Sub-Saharan Africa | 1.16 (1.09, 1.24) | 1.09 (0.96, 1.23) | 1.17 (1.10, 1.25) | 1.10 (0.97, 1.26) | 1.17 (1.10, 1.25) | 1.11 (0.97, 1.28) |
| Latin America | 1.23 (1.16, 1.30) | 1.23 (1.16, 1.32) | 1.23 (1.16, 1.30) | 1.24 (1.16, 1.33) | 1.23 (1.16, 1.30) | 1.24 (1.16, 1.32) |
| Other/Unknown | 1.06 (0.95, 1.18) | 1.19 (1.04, 1.36) | 1.08 (0.97, 1.20) | 1.21 (1.05, 1.39) | 1.07 (0.96, 1.19) | 1.20 (1.04, 1.38) |

MSM: men who have sex with men, IDU: injection drug users; CI: confidence interval; PR: prevalence ratio; LP: late presenters; LPAD: late presenters with advanced disease.

^a^ Adjusted PR (95%CI): adjusted prevalence ratio and 95% CI obtained with multivariable Poisson regression models with robust standard error estimates adjusted for a combined variable of gender and HIV transmission category (MSM, IDU, heterosexual men, heterosexual women and other/unknown), age at enrolment (<30, 30-49, ≥50 years), educational level (None or primary education only, secondary education, university, other/unknown) and region of origin (Europe, Sub-Saharan Africa, Latin America, other/unknown).
