# Supplementary material for: Late presentation for HIV remains a major health issue in Spain: Results from a multicenter cohort study, 2004–2018
Source: PLoS One. 2021 Apr 21;16(4):e0249864. doi: 10.1371/journal.pone.0249864 (PMC8059864; doi:10.1371/journal.pone.0249864)
Supplement: S4 Table — Impact of late presentation and late presentation with advanced disease on the composite endpoint, first AIDS event, first serious non-AIDS event and overall mortality when late presentation and late presentation with advanced disease are defined as participants with an HIV-diagnosis at a CD4 <350 cells/μL (or <200 cells/μL for advanced disease) or with an AIDS-defining event within the 4, 12 or 48 weeks after enrolment. (DOCX) [file pone.0249864.s005.docx]

##### **S4 Table. Impact of late presentation and late presentation with advanced disease on the composite endpoint, first ADE, first SNAE and overall mortality when late presentation and late presentation with advanced disease are defined as participants with an HIV-diagnosis at a CD4 <350 cells/µL (or <200 cells/µL for advanced disease) or with an AIDS-defining event within the 4, 12 or 48 weeks after enrolment**

|  | 1 month |  | 3 months |  | 12 months |  |
| --- | --- | --- | --- | --- | --- | --- |
|  | LP vs non-LP | LPAD vs non-LPAD | LP vs non-LP | LPAD vs non-LPAD | LP vs non-LP | LPAD vs non-LPAD |
|  | Adjusted  IRR (95% CI) ^a, b^ | Adjusted  IRR (95% CI) ^a, b^ | Adjusted  IRR (95% CI) ^a, b^ | Adjusted  IRR (95% CI) ^a, b^ | Adjusted  IRR (95% CI) ^a, b^ | Adjusted  IRR (95% CI) ^a, b^ |
| Composite endpoint ^c^ | 1.29 (1.15, 1.45) | 1.54 (1.36, 1.74) | 1.31 (1.17, 1.47) | 1.57 (1.38, 1.78) | 1.43 (1.27, 1.61) | 1.89 (1.64, 2.17) |
| ADE | 1.29 (1.10, 1.51) | 1.60 (1.33, 1.91) | 1.34 (1.14, 1.58) | 1.65 (1.37, 1.98) | 1.64 (1.39, 1.93) | 2.57 (2.16, 3.05) |
| SNAE ^c, d^ | 1.19 (0.99, 1.43) | 1.43 (1.17, 1.73) | 1.21 (1.00, 1.45) | 1.45 (1.19, 1.76) | 1.24 (1.02, 1.50) | 1.50 (1.21, 1.85) |
| Cardiovascular event | 0.89 (0.62, 1.26) | 0.94 (0.71, 1.24) | 0.87 (0.61, 1.23) | 0.94 (0.71, 1.23) | 0.91 (0.64, 1.29) | 0.94 (0.72, 1.23) |
| Liver event | 1.72 (1.11, 2.66) | 2.12 (1.31, 3.44) | 1.73 (1.11, 2.68) | 2.16 (1.33, 3.51) | 1.72 (1.12, 2.66) | 2.06 (1.28, 3.33) |
| Kidney event | 0.81 (0.23, 2.88) | 1.04 (0.30, 3.58) | 0.83 (0.24, 2.92) | 1.06 (0.31, 3.58) | 0.83 (0.24, 2.89) | 1.04 (0.31, 3.46) |
| Neoplasm | 1.27 (1.05, 1.55) | 1.66 (1.28, 2.15) | 1.32 (1.08, 1.62) | 1.71 (1.32, 2.21) | 1.36 (1.11, 1.67) | 1.82 (1.40, 2.36) |
| Overall mortality | 1.67 (1.36, 2.05) | 1.95 (1.56, 2.44) | 1.70 (1.40, 2.06) | 2.00 (1.61, 2.49) | 1.74 (1.44, 2.11) | 2.10 (1.68, 2.61) |

ADE: AIDS defining event; CI: confidence interval; IRR: incidence rate ratio; LP: late presenters; LPAD: late presenters with advanced disease; PY: person-years; SNAE: serious non-AIDS event

^a^ IRR (CI 95%) were estimated with Poisson regression models with person-years at risk as the offset variable and robust standard error estimates

**^b^** IRR estimates obtained after adjustment for a combined variable of gender and HIV transmission category (MSM, IDU, heterosexual men, heterosexual women and other/unknown), educational level (None or primary education only, secondary education, university, other/unknown), region of origin (Europe, Sub-Saharan Africa, Latin America, other/unknown), and age (<30, 30-49, ≥50 years), presence of HCV antibodies (no, yes or unknown), presence of HBsAg (no, yes or unknown) and viral load (<10,000, 10,000-100,000, ≥ 100,000 copies/mL or unknown) at enrolment.

^c^ Individuals who were monitored in centers not providing data on non-AIDS events were excluded

^d^ The number of specific SNAE do not sum up because one individual can have multiple SNAE
